# Social capital, health behaviours and health: a population-based associational study

**DOI:** 10.1186/1471-2458-13-613

**Published:** 2013-06-27

**Authors:** Tarja Nieminen, Ritva Prättälä, Tuija Martelin, Tommi Härkänen, Markku T Hyyppä, Erkki Alanen, Seppo Koskinen

**Affiliations:** 1 Department of Health, Functional Capacity and Welfare, National Institute for Health and Welfare (THL), Helsinki, Finland; 2 Department of Chronic Disease Prevention, National Institute for Health and Welfare (THL), Turku, Finland

**Keywords:** Individual-level social capital, Social support, Social participation, Trust, Health behaviours, Self-rated health, Psychological well-being

## Abstract

**Background:**

Social capital is associated with health behaviours and health. Our objective was to explore how different dimensions of social capital and health-related behaviours are associated, and whether health behaviours mediate this association between social capital and self-rated health and psychological well-being.

**Methods:**

We used data from the Health 2000 Survey (n=8028) of the adult population in Finland. The response rate varied between 87% (interview) and 77% (the last self-administered questionnaire). Due to item non-response, missing values were replaced using multiple imputation. The associations between three dimensions of social capital (social support, social participation and networks, trust and reciprocity) and five health behaviours (smoking, alcohol use, physical activity, vegetable consumption, sleep) were examined by using logistic regression and controlling for age, gender, education, income and living arrangements. The possible mediating role of health behaviours in the association between social capital and self-rated health and psychological well-being was also analysed with a logistic regression model.

**Results:**

Social participation and networks were associated with all of the health behaviours. High levels of trust and reciprocity were associated with non-smoking and adequate duration of sleep, and high levels of social support with adequate duration of sleep and daily consumption of vegetables. Social support and trust and reciprocity were independently associated with self-rated health and psychological well-being. Part of the association between social participation and networks and health was explained by physical activity.

**Conclusions:**

Irrespective of their social status, people with higher levels of social capital – especially in terms of social participation and networks – engage in healthier behaviours and feel healthier both physically and psychologically.

## Background

Social capital characterises the relations and interactions between individuals and groups. Social capital can be conceptualised and measured at the collective or individual level [1]. Collective social capital is seen to arise in communities and neighbourhoods and is examined as a ‘collective property’. At individual level, social capital is seen as a personal resource that emerges from social networks where individuals have better access to information, services and support. According to the literature, individuals and communities benefit from social capital, for example in the form of better health [2-5].

It has been suggested that social capital affects health through several mechanisms: norms and attitudes that influence health behaviours, psychosocial networks that increase access to health care and psychosocial mechanisms that enhance self esteem [6-8].

Social capital is a multidimensional concept. It includes social networks, social participation and social trust [9]. Social support has sometimes also been considered to be an element of social capital at the individual level [10] but opinions vary on whether it should be included in the concept at all [11-13]. In this article, social support was considered as an indicator of good social relationships and was thus included in the dimensions of social capital.

Health behaviours such as smoking, alcohol consumption, physical activity, dietary choices and duration of sleep are major determinants of health and mortality [14]. Furthermore, they are important determinants of disparities in health between subgroups of the population [15-20]. There is also evidence for an association between social capital and health behaviour [4,8] and social capital and health [4,5]. However, the literature analysing social capital and various health behaviours simultaneously as determinants of health is scarce [21,22].

In a study on the English adult population aged 16 and over, trust, civic participation and support were connected with moderate levels of alcohol consumption [21]. Studies among adults in southern Sweden have indicated that high alcohol consumption is associated with low generalised trust in other people but not with low social participation. The results suggest that high or average social participation in connection with low trust is associated with high alcohol consumption [8,23]. Conversely, an American study found no individual-level association between binge drinking and social participation or trust in one’s community [24].

Studies among adults in Sweden and the United States have shown a positive association between physical activity and social participation [24,25]. The results on diet and social capital are exiguous. Social participation has been found to be positively associated with fruit and vegetable consumption in Sweden [26]. In England, social support, trust and civic participation were positively associated with recommended levels of fruit and vegetable consumption [21]. Few studies have explored the association between the duration of sleep and social capital [4]. In Japan and Britain, social activity was associated with better sleep [27]. In Finland, the level of social capital was associated with daytime vigilance but not with duration of sleep [28].

Findings concerning the potential role of health behaviours as a mediating factor in the association between social capital and health have been inconsistent. An English study found only limited support for this hypothesis [21] while a recent study in the Netherlands [22] concluded that physical activity − but not nutrition, sleeping duration or moderate alcohol intake − acted as a mediating factor in the association between neighbourhood social capital and individual health. Mohnen et al. [22] suggested that the reason for differences between these two studies was that the other study ‘lumped together all available behaviours as mediators…but a separate test of each behaviour might have shown different results’. The authors concluded that future research should also study alternative health outcome variables, such as mental health.

Both of these recommendations have been followed in this paper. Our study includes three dimensions of social capital – covering both structural and cognitive dimensions – and five different health behaviours as well as physical and mental health outcomes. In this paper, we will examine the associations between these at the individual level.

We will focus on the association between social capital and five behaviours − smoking, alcohol consumption, physical activity, consumption of vegetables and duration of sleep − that are associated with health and mortality [29-33]. Low social participation and lack of generalised trust in other people have been reported to be associated with daily smoking in Sweden and England [21,34]. High social capital at work and high levels of social participation and networks have been found to be associated with a greater likelihood of smoking cessation, but in networks of close friends this likelihood decreases [25,35,36].

As mentioned above, it has been suggested that social capital and several socio-demographic factors are associated with health behaviours and health. Also, it has been hypothesised that behaviour might be one possible pathway from social capital to health. However, this hypothesis is still unclear.

Our objective was to explore 1) whether social capital is associated with health behaviours when socio-demographic factors are taken into account and 2) whether social capital is associated with self-rated health and psychological well-being either independently or mediated by health behaviours.

## Methods

### Study population

Our data were derived from the nationally representative Finnish Health 2000 Survey [37,38] (http://www.terveys2000.fi/indexe.html), which was conducted in 2000–2001. The data were collected by means of computer-assisted personal interviews, self-administered questionnaires and a comprehensive clinical health examination. The original sample included 8028 individuals aged 30 or over. The response rate varied between 87% and 77% for the interview and three self-administered questionnaires. The data include a large number of variables that are often used in measuring various aspects of social capital in large population surveys [39,40].

The data were kept highly confidential and ethical issues were considered carefully. The plans and protocols for the Health 2000 Survey were submitted for approval to the relevant ethical committees. Participants were asked for written informed consent. Researchers were required to submit their research plans to the team responsible for the subject area in question in order to obtain expert opinions. Next, the plans were submitted for the review by the project group or its working group to obtain permission to use the data. The data were released to each researcher without access to any personal information [38].

The Health 2000 Survey was approved by the Ethical Committee for Research in Epidemiology and Public Health at the Hospital District of Helsinki and Uusimaa (HUS). The study concerning social capital, health behaviours and health was approved by the project group of the Health 2000 Survey.

### Measures

#### Social capital

We measured social capital with three different dimensions that were based on explorative factor analysis of 36 different variables [40]. These dimensions were *social support* (the belief that emotional support and practical help would be provided when needed), *social participation and networks* (social activities and meeting friends) and *trust and reciprocity* (trust in people, absence of mistrust, feelings of reciprocity, feeling safe in the neighbourhood). In the following, we shorten the names of these three dimensions to support, participation and trust.

Exploratory factor analysis was used to investigate the dimensions of social capital. Available indicators were grouped using three-factor oblique rotation. The number of factors and the rotation method were chosen on the basis of existing theory. The three factors were named 1) social support, 2) social participation and networks, 3) trust and reciprocity. The inter-factor correlations from this model were: support vs. participation (0.28), support vs. trust (0.11) and participation vs. trust (0.04).

Due to the different scales of the indicators, the weighted sums of the indicators within each group were preferred to the unweighted ones in the final operationalisation of the three dimensions of social capital. Applying the one-factor model to each indicator group produced factor scores (weighted sums) with the reliability coefficients 0.90 for support, 0.75 for participation and 0.82 for trust. In the next step, the three factor scores were divided into tertiles. The lowest tertile included persons with low levels of social capital with regard to the dimension in question. Respectively, the upper tertile included those with much social capital.

#### Health behaviour

We analysed five different health behaviours: smoking, use of alcohol, leisure-time physical activity, consumption of vegetables and duration of sleep.

Smoking status was measured by a standard question distinguishing non-smokers (never or occasionally) from smokers (daily). Weekly consumption of alcohol (grams of pure alcohol per week) was calculated on the basis of reported frequency and quantity of drinking. Based on the Finnish Current Care Guideline for treatment of alcohol abuse, heavy drinking was classified as 140 grams or more for women and 280 grams or more for men per week [41]. We dichotomised the use of alcohol as non-excessive drinking vs. excessive drinking. Non-excessive drinkers also include abstainers: nearly 12% of men and 23% of women stated they had not drunk any alcohol during the past 12 months.

Consumption of vegetables was based on the question: ‘How often have you eaten vegetables during the last week?’ The alternative answers were ‘never’, ‘once or twice’, ‘3−5 times’ and ‘6−7 times’. The responses were dichotomised into daily (6–7 times a week) consumption of vegetables vs. less.

Leisure-time physical activity was based on a question ‘How much do you exercise and strain yourself physically in your leisure-time?’ with four alternative responses: sedentary, light, moderate and competitive sport. They were dichotomized to ‘sedentary’ and ‘active’. ‘Active’ includes all of the other three alternatives. Duration of sleep was asked as hours of sleep per 24 hours. It was dichotomized as adequate sleep (7–8 hours in 24 hours), and less or more.

#### Self-rated health and psychological well-being

We used self-rated health and psychological well-being as health indicators. The respondents were asked to rate their own health on a five-item scale. The answers were dichotomised to good (good or rather good) and poor (average, rather poor or poor) health. Psychological well-being was based on the 12-item General Household Questionnaire (GHQ12) with a cut-point of 2/3, where 0–2 indicates psychological well-being (lack of psychological distress). The GHQ12 total score was calculated only if at least ten questions had been answered. When one or two values were missing, they were substituted with the average of the other items.

#### Socio-demographic factors

Five socio-demographic factors were included in the analysis as covariates: gender, age group, education, living arrangements and household income. *Age* was classified into six categories: 30–39, 40–49, 50–59, 60–69, 70–79, and 80 or more years. *Education* was based on register data on the highest educational degree completed by the respondents and it included three groups: basic (no matriculation examination or vocational training), secondary (matriculation examination or completed vocational school) and higher (degree from a higher vocational institution, polytechnic or university). *Living arrangements* were categorised into four groups based on official marital status and household composition: married, cohabiting, living with persons other than a partner (for example with children or siblings) and living alone. *Income* was based on register information on the monthly income of the household divided by the number of consumption units, where the first adult of the household was assigned a value of 1, other adults a value of 0.7 and children a value of 0.5. Income per consumption unit (1000 euros) was included in the analyses as a continuous variable. The income distribution was right-censored at 200 in order to obtain numerical stability in the estimation procedures. The six highest outliers whose income values were over 200 were assigned a value of 200 in the analyses.

### Data analysis

Non-response was greater in the self-administered questionnaires than in the interview. Due to higher item non-response in the case of trust and duration of sleep compared to the other variables, a complete-case analysis would have resulted in the loss of much information (see Table 1). Therefore we decided to replace the missing values using multiple imputation [42]. The MCMC method of the MI procedure of the SAS System [43] was applied to create 50 imputed datasets concerning the respondents. The variables in the imputation model were support, participation, trust, age, gender, education, living arrangements, income, smoking, drinking, physical activity, vegetable consumption, duration of sleep, income and self-rated and psychological health.

**Table 1:** The percentage of missing data (PM), and the weighted prevalences (%) based on the multiply imputed data for all variables used in further analyses

**VARIABLES**	**All**		**Men**		**Women**	
	**PM**	**%**	**N**	**%**	**N**	**%**
Smoking	8		3311		4046	
Daily		23		29		17
Never/occasionally		77		71		83
Drinking	16		3007		3754	
Excessive		9		13		5
Non-excessive		91		87		95
Leisure-time physical activity	18		2959		3641	
Sedentary		29		28		29
Active		71		72		71
Use of vegetables	12		3150		3897	
Less than daily		43		49		37
Daily		57		51		63
Sleeping duration	25		2642		3344	
Less/more than 7-8h		29		28		30
7–8 hours		71		72		70
Self-rated health	8		3305		4059	
Poor		39		39		39
Good		61		61		61
Psychological well-being	18		2939		3606	
Poor		25		23		27
Good		75		77		73
Gender	0		3637		4391	
Men		47				
Women		53				
Age	0		3637		4391	
30-39		22		24		21
40-49		24		25		22
50-59		23		24		21
60-69		15		15		15
70-79		11		9		13
80-		5		3		8
Education	0		3637		4391	
Basic		40		39		41
Secondary		33		35		31
Higher		27		26		28
Living arrangements	10		3282		3970	
Married		57		62		54
Cohabiting		11		12		10
Living with other(s)		8		6		9
Living alone		24		20		27
Support	22		2816		3452	
Low		38		43		33
Moderate		32		33		32
High		30		24		35
Participation	24		2761		3337	
Low		33		42		25
Moderate		35		35		35
High		32		23		40
Trust	30		2539		3047	
Low		33		28		36
Moderate		36		35		37
High		31		37		27

Statistical analyses were performed on each imputed dataset with Sudaan, which takes into account the complex sampling design – that is, stratification, clustering and sampling weights based on poststratification [44,45] – and finally pools the results corresponding to the 50 data-sets adjusting the standard errors appropriately [42]. We used logistic regression to assess the association of social capital and socio-demographic factors with non-smoking status, non-excessive drinking, leisure-time physical activity, daily consumption of vegetables and adequate duration of sleep.

We elaborate the association between social capital and each health behaviour (Table 2). Model M2a presents the association between the dimensions of social capital separately for each health behaviour, adjusting for gender and age group. In Model 2b, we include all the dimensions of social capital simultaneously, adjusting for gender and age group. Then we add all the socio-demographic factors (education, living arrangements and income) simultaneously with all the earlier variables (Model 2c).

**Table 2:** Associations between social capital and healthy behaviour patterns (odds ratios with 95% CI for Model M2c)

**Variables**	**M2a: age +gender + var**^**1**^	**M2b: age +gender +social capital**^**2**^	**M2c: M2b+ socio-demographic factors**^**3**^	**CI (95 %)**^**4**^
**NON-SMOKING**				
Social support				
Low	1.00	1.00	1.00	
Moderate	1.10	0.96	0.84 *	(0.72-0.99)
High	1.47 ***	1.13	0.88	(0.74-1.04)
Social participation and networks				
Low	1.00	1.00	1.00	
Moderate	1.56 ***	1.51 ***	1.41 ***	(1.20-1.65)
High	2.80 ***	2.66 ***	2.34 ***	(1.97-2.78)
Trust and reciprocity				
Low	1.00	1.00	1.00	
Moderate	1.25 **	1.20 *	1.09	(0.94-1.27)
High	1.67 ***	1.56 ***	1.33 **	(1.13-1.58)
**NON-EXCESSIVE DRINKING**				
Social support				
Low	1.00	1.00	1.00	
Moderate	0.98	0.89	0.87	(0.69-1.08)
High	0.98	0.82	0.79	(0.61-1.00)
Social participation and networks				
Low	1.00	1.00	1.00	
Moderate	1.37 **	1.39 **	1.44 **	(1.15-1.79)
High	1.87 ***	1.93 ***	2.01 ***	(1.53-2.61)
Trust and reciprocity				
Low	1.00	1.00	1.00	
Moderate	1.11	1.10	1.07	(0.85-1.33)
High	1.25	1.22	1.16	(0.91-1.46)
**LEISURE-TIME PHYSICAL ACTIVITY**				
Social support				
Low	1.00	1.00	1.00	
Moderate	1.23 **	1.05	1.02	(0.89-1.18)
High	1.55 ***	1.11	1.04	(0.89-1.23)
Social participation and networks				
Low	1.00	1.00	1.00	
Moderate	2.46 ***	2.43 ***	2.40 ***	(2.10-2.75)
High	4.94 ***	4.83 ***	4.73 ***	(3.97-5.62)
Trust and reciprocity				
Low	1.00	1.00	1.00	
Moderate	0.99	0.92	0.91	(0.78-1.05)
High	1.18 *	1.06	1.02	(0.87-1.20)
**DAILY USE OF VEGETABLES**				
Social support				
Low	1.00	1.00	1.00	
Moderate	1.31 ***	1.22 **	1.12	(0.99-1.27)
High	1.72 ***	1.47 ***	1.25 **	(1.09-1.454)
Social participation and networks				
Low	1.00	1.00	1.00	
Moderate	1.43 ***	1.37 ***	1.31 ***	(1.15-1.49)
High	2.20 ***	2.02 ***	1.85 ***	(1.61-2.11)
Trust and reciprocity				
Low	1.00	1.00	1.00	
Moderate	1.05	1.00	0.95	(0.83-1.07)
High	1.18 *	1.08	0.98	(0.85-1.14)
**ADEQUATE DURATION OF SLEEP**				
Social support				
Low	1.00	1.00	1.00	
Moderate	1.26 **	1.17 *	1.11	(0.96-1.29)
High	1.59 ***	1.39 ***	1.24 **	(1.06-1.46)
Social participation and networks				
Low	1.00	1.00	1.00	
Moderate	1.34 ***	1.28 ***	1.24 **	(1.08-1.42)
High	1.65 ***	1.50 ***	1.42 ***	(1.20-1.67)
Trust and reciprocity				
Low	1.00	1.00	1.00	
Moderate	1.36 ***	1.31 **	1.27 **	(1.09-1.48)
High	1.47 ***	1.38 ***	1.30 **	(1.12-1.52)

Health 2000 Survey, adults 30+ years (N=8028).

*** p<0.001, ** p<0.01, * p<0.05.

^1^ Model 2a includes age, gender and one dimension of social capital at a time.

^2^ Model 2b includes age, gender and all dimensions of social capital (social support, social participation and networks, and trust and reciprocity) simultaneously.

^3^ Model 2c includes age, gender, all the dimensions of social capital, all the socio-demographic factors (education, living arrangements and income) simultaneously.

^4^ CI confidence intervals for Model 2c.

Interactions between dimensions of social capital and socio-demographic factors (age, gender, education, living arrangements, income) were also analysed to find out whether the associations between the dimensions of social capital and health behaviours are similar in different subgroups of the population. These interaction estimates and the corresponding p-values were based on the original dataset without imputation, because the SAS version 9.1 did not provide adequate tools to implement interactions in the imputation model.

The mediating effects of health behaviour between social capital and health was assessed by analysing the contribution of five health behaviours to differences in self-rated health (Table 3) and psychological well-being (Table 4) between the levels of social capital. Models 3a (Table 3) and 4a (Table 4) include all dimensions of social capital and socio-demographic factors simultaneously, adjusted for age and gender. Then all the health behaviours were added one by one (Models 3b-f and Models 4b-f). Finally, we added all of them simultaneously (Models 3g and 4g).

**Table 3:** The contribution of health behaviours to the association between social capital and self-rated health (odds ratios with 95% CI for Model 3g)

**Social capital**	**M3a**^**1**^	**M3b: M3a + smoking**^**2**^	**M3c: M3a + drinking**^**2**^	**M3d: M3a + physical activity**^**2**^	**M3e: M3a + vegetables**^**2**^	**M3f: M3a + sleep**^**2**^	**M3g: M3a + all health behaviours**^**3**^	**CI 95%**^**4**^
**SUPPORT**								
Low	1.00	1.00	1.00	1.00	1.00	1.00	1.00	
Moderate	1.16*	1.16*	1.16*	1.16*	1.15	1.15	1.15	1.00-1.33
High	1.26**	1.26**	1.26**	1.25**	1.25**	1.24**	1.23*	1.05-1.44
**PARTICIPATION**								
Low	1.00	1.00	1.00	1.00	1.00	1.00	1.00	
Moderate	1.41***	1.39***	1.40***	1.26**	1.39***	1.39***	1.23*	1.05-1.43
High	1.86***	1.81***	1.84***	1.56***	1.81***	1.81***	1.49***	1.26-1.75
**TRUST**								
Low	1.00	1.00	1.00	1.00	1.00	1.00	1.00	
Moderate	1.30***	1.30***	1.30***	1.33***	1.31***	1.28**	1.31***	1.13-1.51
High	2.11***	2.09***	2.11***	2.13***	2.11***	2.08***	2.09***	1.76-2.48

Health 2000 Survey, adults 30+ years (N=8028).

^1^ Model 3a includes age, gender, all dimensions of social capital (social support, social participation and networks, and trust and reciprocity) and socio-demographic factors (education, living arrangements and income) simultaneously.

^2^ Models 3b-f include Model 3a and b) smoking, c) drinking, d) physical activity, e) vegetable consumption and f) duration of sleep.

^3^ Model 3g includes Model 3a and all health behaviours (b-f) simultaneously.

^4^ CI confidence intervals for Model 3g.

**Table 4:** **The contribution of health behaviours to the association between social capital and psychological well-being (odds ratios with 95**% **CI for Model 4g)**

**Social capital**	**M4a**^**1**^	**M4b: M4a+smoking**^**2**^	**M4c: M4a+drinking**^**2**^	**M4d: M4a+physical activity**^**2**^	**M4e: M4a+vegetables**^**2**^	**M4f: M4a+sleep**^**2**^	**M4g: M4a+all health behaviours**^**3**^	**CI 95%**^**4**^
**SUPPORT**								
Low	1.00	1.00	1.00	1.00	1.00	1.00	1.00	
Moderate	0.99	0.99	0.99	0.99	0.98	0.98	0.98	0.85-1.13
High	1.11	1.11	1.12	1.11	1.10	1.09	1.09	0.91-1.31
**PARTICIPATION**								
Low	1.00	1.00	1.00	1.00	1.00	1.00	1.00	
Moderate	1.26**	1.26**	1.25**	1.17*	1.25**	1.24**	1.14	0.98-1.33
High	1.38***	1.37***	1.34***	1.22*	1.35***	1.34***	1.16	0.99-1.37
**TRUST**								
Low	1.00	1.00	1.00	1.00	1.00	1.00	1.00	
Moderate	2.01***	2.01***	2.01***	2.04***	2.02***	1.98***	2.01***	1.72-2.35
High	3.91***	3.90***	3.91***	3.93***	3.92***	3.85***	3.89***	3.28-4.61

Health 2000 Survey, adults 30+ years (N=8028).

^1^ Model 4a includes age, gender, all dimensions of social capital (social support, social participation and networks, and trust and reciprocity) and socio-demographic factors (education, living arrangements and income) simultaneously.

^2^ Models 4b-f include Model 4a and b) smoking, c) drinking, d) physical activity, e) vegetable consumption and f) duration of sleep.

^3^ Model 4g includes Model 4a and all health behaviours (b-f) simultaneously.

^4^ CI confidence intervals for Model 4g.

## Results

Table 1 presents the distribution of each health behaviour, socio-demographic characteristics and the three dimensions of social capital by gender.

The only dimension of social capital that was clearly associated with all types of health behaviour was participation (Table 2). These associations were statistically significant even after controlling for the other dimensions of social capital and socio-demographic characteristics. There was also a clear gradient: the higher the level of participation, the greater the odds for healthy behaviour, especially when it comes to leisure-time physical activity.

Support was positively associated with consumption of vegetables and duration of sleep. When the other dimensions of social capital, education and living arrangements had been adjusted for, only the highest level of support was associated with these two health behaviours. High levels of support were also associated with non-smoking and leisure-time physical activity, but when the other dimensions of social capital were added to the model, this association disappeared. Social support did not seem to have any association with drinking.

High levels of trust were associated with non-smoking and adequate duration of sleep, and also to a slight extent with non-excessive drinking and daily consumption of vegetables, before adjusting for the other variables. Trust was not associated with leisure-time physical activity. The associations between trust and non-smoking and adequate duration of sleep remained statistically significant after all the variables were added to the model.

Two interactions were found by gender: between sleep and support and between physical activity and participation. The gradient of these associations runs in the same direction in both genders but was steeper among men in the case of sleep and among women in the case of physical activity. The interaction of gender and support based on the data set without imputation was statistically significant (p-value 0.021), and the OR estimate for men (high vs. low support) was 1.94 and for women 1.35, respectively. The interaction of gender and participation was statistically significant (p = 0.049), and the OR estimate for men (high vs. low support) was 3.38 and for women 5.16, respectively.

Also, the association between physical activity and participation varied according to age. Low levels of participation were associated with physical inactivity in all age groups. However, unlike the other age groups, in those 60–69 years of age, moderate levels of participation were associated with physical activity as much as high levels of participation.

The association of social capital with health behaviours was similar in all the categories of living arrangements and almost similar in the education categories. Low levels of social capital were associated with unhealthy behaviours regardless of educational level and living arrangements. As no systematic variation could be recognized, we analysed all the subgroups together.

High levels of support (OR 1.26), participation (OR 1.86) and trust (OR 2.11) were associated with good self-rated health when socio-demographic factors were taken into account (Table 3). The association between all the dimensions of social capital and self-rated health remained constant regardless of smoking, drinking, vegetable consumption or duration of sleep. The only exception was physical activity, which reduced the independent association between participation and self-rated health by about 36%.

High levels of participation were associated with psychological well-being (OR 1.38 for high versus low participation, Table 4); health behaviours did not contribute to this association, with the exception of physical activity, which reduced the OR to 1.22. Those with high levels of trust reported that their health was good about four times more often than those with low levels of trust. Health behaviours did not change this association. Support was not associated with psychological well-being after adjusting for the other dimensions of social capital (Table 4).

## Discussion

This study on social capital, health behaviours and health among Finnish adults showed that social participation was the only dimension of social capital clearly associated with all types of health behaviours irrespective of socio-demographic characteristics. We also found that health behaviours did not explain the association between social capital and health, except for physical activity, which moderately attenuated the association of participation with both self-rated health and psychological well-being.

### Social capital and health behaviours

High levels of social participation have been suggested to be associated with smoking cessation and active leisure-time physical activity but the results concerning alcohol consumption have been inconsistent [8].

We found that active social participation was significantly associated with non-smoking, non-excessive drinking, leisure-time physical activity, daily consumption of vegetables and adequate duration of sleep. Controlling for socio-demographic factors attenuated these associations only slightly. The observed associations between social participation, non-smoking and daily consumption of vegetables confirm earlier findings [26,34]. Social participation was the only dimension of social capital that was associated with consumption of alcohol when controlling for other factors: the higher the level of social participation, the greater the likelihood of non-excessive drinking. It might be that the norms of social networks control excessive alcohol consumption. People enjoy being together if drinking remains moderate. On the other hand, it has been suggested that active participation coupled with low trust results in greater alcohol consumption [23].

One can argue that chronic diseases reduce opportunities to participate in social activities and establish networks. We checked whether chronic diseases explain the association between participation and leisure-time physical activity. However, having one or more chronic diseases did not essentially change the odds for participation: social participation still had a strong independent association with leisure-time physical activity.

People with high levels of social support tended to consume more vegetables and sleep adequately. It has been suggested that supportive social relationships can reduce the probability of individuals adopting unhealthy behaviours by minimising the impact of daily stressors or stressful events [46]. Our study did not support this observation, as social support was associated with only two of the five studied health behaviours. Previous findings have shown that having a spouse and/or supportive family and friends is positively associated with increased physical activity [47]. Our results suggest that social participation explains this association. After controlling for social participation, social support and living arrangements were no longer associated with physical activity.

According to our analyses, all dimensions of social capital were positively associated with adequate duration of sleep. Common sense would seem to suggest that your sleep is more relaxed if you trust people and have support in stressful situations. When you are socially active, you feel appreciated by other people, and an active lifestyle makes it easier to fall asleep. Duration of sleep was the only health behaviour included in this study that was associated with all dimensions of social capital after controlling for education, living arrangements and income. Associations between social support [48] and leisure-time activity [27] and the quality of sleep have been reported earlier but as far as we know there are very few reports about the association of social capital and duration of sleep. In contrast to our findings on the association between the individual level social capital and sleep, sleep duration was not affected by the level of social capital in the neighbourhood [22].

Unlike the other dimensions of social capital, social support was not associated with non-smoking. This seems to be consistent with earlier studies [36,49]. However, it has been suggested that high social participation combined with a low level of trust has an adverse association with smoking [34]. Studies have suggested that adolescents who are provided with more emotional and practical help would gain buffers towards stress, making it easier for them not to smoke [49]. Although our measure of social support included both emotional and practical help, it was not associated with smoking among adults when other dimensions of social capital were considered. One explanation could be that social support is important in youth when health behaviours are still developing; in adulthood, all habits including health behaviours have already become more stable.

The reasons for the strong association between social participation and health behaviours remain vague and challenging to verify. It might be that social participation and networks give one a greater feeling of belonging. In networks, people can meet others who are different from them – the kinds of persons they might not meet otherwise. Networks with a diverse range of members might provide good role models and information on healthier behaviour. Social desirability might also make one more willing to accept new ideas. On the other hand, inactive people might stay at home more often and fill their emptiness and loneliness with alcohol and unhealthy food, for example, which might lead to an even less active life.

The interaction between social participation and physical activity suggested more apparent association among women than among men. According to focus interviews [50], the central motivators towards physical activity are to keep fit and to meet with friends and that way also keep motivated. As interpersonal relationships may be more central to women than to men, social participation might promote physical activity especially among women. However, no further analysis was undertaken as this was not the main scope of the study.

### Health behaviours as mediators in the association between social capital and health

In our study, all dimensions of social capital were independently associated with self-rated health while social participation and trust (but not support) were associated with psychological well-being. The only health behaviour that mediated this association was leisure-time physical activity, which contributed to the association between social participation and both self-rated health and psychological well-being, supporting the findings of a recent Dutch study [22]. Based on this cross-sectional study, it is not possible to say whether social capital influences physical activity or vice versa. However, both physical activity and social participation are associated with better health.

Social capital and health behaviours are both associated with self-rated health. As we examined an subjective measure of health, social capital might affect the feeling of well-being through psychosocial mechanisms. Health behaviours are known to affect health, but their effects might only become evident in the long term.

The effect of social capital on health has been repeatedly proposed to be mediated through health behaviours [4]. The recent findings suggest similar results with this study that physical activity is mediating the influence of social capital on health: the direct effect of social capital on health becomes weaker if physical activity is included in the model [22]. However, these mediating associations still remain debatable.

### Methodological considerations

To verify the random nature of the missing data in our original dataset, we performed sensitivity analyses based on two opposite scenarios. Either all missing social capital and health behaviour variables were set to the most positive values or, in the other scenario, to the most negative values. The conclusions and the statistical significances based on these scenarios were practically the same as with the original data, but the estimated associations were slightly weaker.

In the case of trust and duration of sleep, item non-response was higher than for the other variables. Therefore a multiple imputation method was employed to substitute the missing data. This method decreased the standard errors of the log OR estimates by approximately 10% (data not shown) when compared to a corresponding complete case analysis. However, the imputations did not alter the conclusions.

The association between participation and leisure-time physical activity proved to be particularly strong and, consequently, physical activity contributed to the association between participation and health. These findings may, however, partly result from the fact that our measure of participation is based on a battery of questions that included one question on exercising, hunting, fishing, gardening and other outdoor activities. Therefore, we repeated the analyses without this question, but this only slightly attenuated the strength of the association between participation and leisure-time physical activity, and thus it remained the most evident association.

The strength of this study is that it is based on a nationally representative population sample taken from the Health 2000 Survey in Finland [38]. Also, we used multiple indicators of structural and cognitive social capital at the individual level [see for [4,5,9]. Social capital is often considered to be a contextual resource. It would be valuable to analyse social capital at both the individual and contextual levels simultaneously. In the absence of community-level contextual indicators, we measured associations of social capital at the individual level. Also, we did not aggregate individual-level indicators to contextual level, and furthermore, we did not employ a multilevel modelling technique in order to separate individual and contextual effects, but we separated the individual-related social support from the individual-level social participation proxy. It has been previously suggested that leisure-time social participation, representing the structural dimension of social capital, is a stable feature that is applicable in long-term epidemiological surveys [51]. We measured several health behaviours, including sleep duration that did not explain the effects of neighbourhood-level social capital on health in Netherlands [22]. The cross-sectional setting of our study does not allow causal inferences. As the next step, longitudinal data will be collected to test the possible reversed causality. Also, it is unlikely that our study considered all potential confounders. Hence, residual confounding is possible and could contribute to the observed associations.

## Conclusions

People with high levels of social participation and networks have healthier behaviours. Although health-related behaviour does not explain the association between the individual-level social capital and health, there might be other mechanisms that do. Longitudinal epidemiological studies will be needed to examine and to prove the significance of social capital in health promotion.

## Competing interests

The authors declare that they have no competing interests.

## Authors’ contributions

TN processed the data, carried out the statistical analyses, drafted and finalised the manuscript. RP, TM, MTH and SK supervised the first author and participated in conception, designing and interpreting the data and drafting the manuscript. TH and EA provided advice on the statistical analyses. All authors revised the text critically for important intellectual content and read and approved the final manuscript.

## Pre-publication history

The pre-publication history for this paper can be accessed here:

http://www.biomedcentral.com/1471-2458/13/613/prepub

## References

[B1] De Silva MJ McKenzie K Harpham T Huttly SR Social capital and mental illness: a systematic review J Epidemiology and Community Health 2005 59 619 627 10.1136/jech.2004.029678 PMC1733100 16020636

[B2] Mohnen SM Groenewegen PP Völker B Flap H Neighbourhood social capital and individual health Soc Sci Med 2011 72 5 660 667 10.1016/j.socscimed.2010.12.004 21251743

[B3] Giordano GN Henrik O Lindström M Social capital and health:Purely a question of context? Health Place 2011 17 946 953 10.1016/j.healthplace.2011.04.004 21555233

[B4] Hyyppa MT Healthy ties. Social capital, population health and survival 2010 Berlin-Heidelberg-New York: Springer

[B5] Kawachi I Subramanian SV Kim D Social capital and health 2008 New York; London: Springer

[B6] Kawachi I Kennedy B Glass R Social capital and self-rated health: a contextual analysis Am J Public Health 1999 89 8 1187 1193 10.2105/AJPH.89.8.1187 10432904 PMC1508687

[B7] Kawachi I Berkman L Social cohesion, social capital and health Social Epidemiology 2000 Oxford: Oxford University Press 174 190

[B8] Lindström M Kawaci I, Subramanian S, Kim D Social capital and health-related behaviors Social capital and health 2008 New York: Springer Science + Business Media, LLC 215 238

[B9] Macinko J Starfield B The utility of social capital in research on health determinants Milbank Q 2001 79 3 387 427 10.1111/1468-0009.00213 11565162 PMC2751199

[B10] Zukewich N Norris D National experiences and international harmonization in social capital measurement: a beginning 2005 Helsinki: In Siena Group meeting

[B11] Lochner K Kawachi I Kennedy B Social capital: a guide to its measurement Health Place 1999 5 4 259 270 10.1016/S1353-8292(99)00016-7 10984580

[B12] Portes A Social Capital: Its Origins and Applications in Modern Sociology Annual Review of Sociology 1998 24 1 24 10.1146/annurev.soc.24.1.1

[B13] McOrmond T Babb P Conceptualising and defining social capital with a policy relevant focus In *Siena Group meeting in Finland* 2005 URL:http://www.stat.fi/sienagroup2005/trish.pdf

[B14] Commission E The state of health in the European Community: report from the Commission 1996 Luxembourg: European Commission

[B15] Laaksonen M Interrelationships among daily health behaviours : towards health-related lifestyle 2002 Helsinki: National Public Health Institute

[B16] Palosuo H Koskinen S Lahelma E Kostiainen E Prättälä R Martelin T Ostamo A Keskimäki I Sihto M Linnanmäki E Health inequalities in Finland. Trends in socioeconomic health differences 1980–2005. Volume 9 2009 Helsinki: Ministry of Social Affairs and Health Publications

[B17] Giskes K Kunst AE Benach J Borrell C Costa G Dahl E Dalstra JA Federico B Helmert U Judge K Trends in smoking behaviour between 1985 and 2000 in nine European countries by education J Epidemiol Community Health 2005 59 5 395 401 10.1136/jech.2004.025684 15831689 PMC1733079

[B18] Prättälä R Hakala S Roskam AJ Roos E Helmert U Klumbiene J Van Oyen H Regidor E Kunst AE Association between educational level and vegetable use in nine European countries Public Health Nutr 2009 12 11 2174 2182 10.1017/S136898000900559X 19402946

[B19] Varo JJ Martínez-González MA De Irala-Estévez J Kearney J Gibney M Martínez JA Distribution and determinants of sedetary lifestyles in the European Union Int J Epidemiol 2003 32 1 138 146 10.1093/ije/dyg116 12690026

[B20] Mackenbach JP Bakker M Reducing inequalities in health 2002 London: Routledge

[B21] Poortinga W Do health behaviours mediate the association between social capital and health? Prev Med 2006 43 488 493 10.1016/j.ypmed.2006.06.004 16860857

[B22] Mohnen SM Völker B Flap H Groenewegen PP Health-related bahvior as a mechanism behind the relationship between neighborhood social capial and individual health - a multilevel analysis BMC Public Health 2012 12 116 10.1186/1471-2458-12-116 22325740 PMC3347984

[B23] Lindström M Social capital, the miniaturization of community and high alcohol consumption: A population-based study Alcohol Alcohol 2005 40 6 556 562 10.1093/alcalc/agh190 16087659

[B24] Greiner K Chaoyang L Kawachi I Hunt D Ahluwalia J The relationships of social participation and community ratings to health behaviors in areas with high and low population density Soc Sci Med 2004 59 2303 2312 10.1016/j.socscimed.2004.03.023 15450705

[B25] Lindström M Moghaddassi M Merlo J Social capital and leisure time physical activity: a population based multilevel analysis in Malmö, Sweden J Epidemiology and Community Health 2003 57 23 28 10.1136/jech.57.1.23 PMC1732271 12490644

[B26] Lindström M Hanson B Wirfält E Östergren P Socioeconomic differences in the consumption of vegetables, fruit and fruit juices: The influence of psychosocial factors Eur J Public Health 2001 11 51 59 10.1093/eurpub/11.1.51 11276572

[B27] Nasermoaddeli A Sekine M Kumari M Chandola T Marmot M Kagamimori S Association of sleep quality and free time leisure activities in Japanese and British civil servants J Occup Health 2005 47 384 390 10.1539/joh.47.384 16230831

[B28] Hyyppä M Kronholm E Kansalaisyhteisön sosiaalinen pääoma heijastuu vireyteen Suom Laakaril 2002 57 41 4124 4127

[B29] Peto R Lopez AD Boreham J Thun M Heath C Jr Doll R Mortality from smoking worldwide Br Med Bull 1996 2 1 12 21 8746293 10.1093/oxfordjournals.bmb.a011519

[B30] White IR Altmann DR Nanchahal K Alcohol consumption and mortality: modelling risks for men and women at different ages BMJ 2002 325 7357 191 197 10.1136/bmj.325.7357.191 12142306 PMC117446

[B31] Byberg L Melhus H Gedeborg R Sundström J Ahlbom A Zethelius B Berglund LG Wolk A Michaëlsson K Total mortality after changes in leisure time physical activity in 50 year old men: 35 year follow-up of population based cohort Br J Sports Med 2009 43 7 482 489 19581403

[B32] Khaw K-T Wareham N Bingham S Welch A Luben R Day N Combined impact of health behaviours and mortality in men and women: the EPIC-Norfolk prospective population study PLoS Med 2008 5 39 47 10.1371/journal.pmed.0050039 PMC2174962 18184033

[B33] Hublin C Partinen M Koskenvuo M Kaprio J Sleep and mortality: a population-based 22-year follow-up study Sleep 2007 30 10 1245 1253 17969458 10.1093/sleep/30.10.1245 PMC2266277

[B34] Lindström M Social capital and miniaturization of community among daily smokers and intermittent smokers: a population-based study Prev Med 2003 36 177 184 10.1016/S0091-7435(02)00049-X 12590993

[B35] Kouvonen A Oksanen T Vahtera J Väänänen A De Vogli R Elovainio M Pentti J Leka S Cox T Kivimäki M Work-place social capital and smoking cessation: the Finnish Public Sector Study Addiction 2008 103 11 1857 1865 10.1111/j.1360-0443.2008.02315.x 18705683

[B36] Lindström M Isacsson S Elmstahl S Impact of different aspects of social participation and social capital on smoking cessation among daily smokers: a longitudinal study Tob Control 2003 12 3 274 281 10.1136/tc.12.3.274 12958387 PMC1747740

[B37] Aromaa A Koskinen S Health and functional capacity in Finland. Baseline results of the Health 2000 health examination survey 2004 Helsinki: National Public Health Institute

[B38] Heistaro S Methodology report, Health 2000 Survey 2008 Helsinki: Publications of the National Public Health Institute B26

[B39] Sosiaalinen pääoma suomalaisissa haastattelu- ja kyselyaineistoissa vuoden 1990 jälkeen. (in Finnish). Social capital in the Finnish surveys after year 1990 http://www.stat.fi/org/tut/dthemes/papers/sospaaoma_sospaa.html

[B40] Nieminen T Martelin T Koskinen S Simpura J Alanen E Härkänen T Aromaa A Measurement and socio-demographic variation of social capital in a large population-based survey Social Indicators Research 2008 85 3 405 423

[B41] Current Care Summary, tFMSD Working group appointed by the Finnish Society of Addiction Medicine Treatment of alcohol abuse 2010 URL: http://www.kaypahoito.fi/web/kh/suositukset/naytaartikkeli/tunnut/ccs00005

[B42] Rubin DB Multiple Imputation for Nonresponse in Surveys 1987 New York: John Wiley & Sons, Inc.

[B43] SAS Institute Inc SAS/STAT® 9.2 User’s Guide 2009 Second Cary, NC: SAS Institute Inc

[B44] Research Triangle Institute SUDAAN User's Manual, Release 8.0 2001 Research Triangle Park, NC: Research Triangle Institute

[B45] Djerf K Laiho J Lehtonen R Härkänen T Knekt P Heistaro S Weighting and statistical analysis Methodology report, Health 2000 Survey. Volume B26 2008 Helsinki: Publications of the National Public Health Institute 182 200

[B46] Berkman L Glass T Berkman L, Kawachi I Social integration, social networks, social support, and health Social epidemiology 2000 New York: Oxford University Press 137 173

[B47] Eyler A Brownson R Donatelle R King A Brown D Sallis J Physical activity social support and middle- and older-aged minority women: results from a US survey Soc Sci Med 1999 49 781 789 10.1016/S0277-9536(99)00137-9 10459889

[B48] Nordin M Knutson A Sundbom E Is disturbed sleep a mediator in the association between social support and myocardial infarction? J Health Psychol 2008 13 55 64 10.1177/1359105307084312 18086718

[B49] Lundborg P Social capital and substance use among Swedish adolecents - an explorative study Soc Sci Med 2005 61 6 1151 1158 10.1016/j.socscimed.2004.12.031 15970227

[B50] EaS D Public attitudes to physical activity in 2011 Report for Glasgow Centre for Population Health: Glasgow

[B51] Hyyppa MT Mäki J Alanen E Impivaara O Aromaa A Long-term stability of social participation Social Indicators Research 2008 88 389 396 10.1007/s11205-007-9199-y

